# Intestinal Obstruction of Congenital Origin: A Case Report

**DOI:** 10.31729/jnma.4708

**Published:** 2020-01-31

**Authors:** Mandeep Guragai, Suzit Bhusai, Anwesh Bhatta

**Affiliations:** 1 Kathmandu Medical College and Teaching Hospital, Sinamangai, Kathmandu, Nepal; 2 Department of Paediatrics, Kathmandu Medical College and Teaching Hospital, Sinamangal, Kathmandu, Nepal

**Keywords:** *case reports*, *congenital abnormalities*, *intestinal obstruction*, *vomiting*

## Abstract

Congenital bands are rare causes of intestinal obstruction and often leads to diagnostic challenges. Diagnostic delays in cases of mechanical obstruction might lead to irreversible bowel ischemia and perforation. Presently described is a case of an 18 month young child with severe vomiting developed for one day. The child was initially thought to have acute viral enteritis and treated accordingly. Due to the severity, an X-Ray and computed tomography scan were sent which pointed towards the possibility of having congenital bands. He was treated operatively. The child was kept under observation for eleven days and was discharged. Although rare, intestinal obstruction due to congenital bands must be considered when treating a child with severe vomiting.

## INTRODUCTION

Obstruction in the gastrointestinal tract of a child can occur anywhere from the mouth to the anus.^1^ Congenital bands have not been thoroughly evaluated in the literature and their etiologies are obscure while some suggest them to be a mesenteric anomaly.^2^ Although congenital bands that lead to intestinal obstruction are very rare,^3^ a delay in the diagnosis could lead to irreversible bowel ischemia and perforation and presents a surgical emergency.^4^

## CASE REPORT

An 18 month young male child from Kathmandu presented to the emergency department of our hospital with complaints of multiple episodes of severe vomiting since one day. The vomitus was bilious, projectile in nature and occurred after every meal. According to his father, the child had had his dinner the preceding night. After a few hours of sleep, the child started vomiting and was then brought to the hospital the following morning when it didn't stop throughout the night. Ondansetron 1ml was immediately given to the child orally. An intravenous line was opened and 200ml of normal saline was infused. Paediatric consultation was done and the child was admitted and shifted to the paediatric ward.

**Figure 1.**
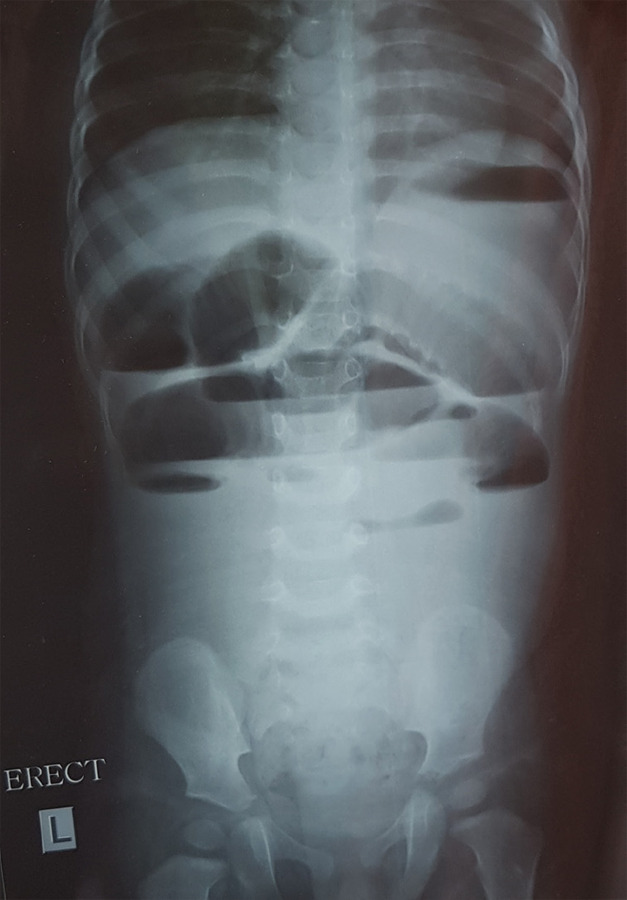
Plain radiograph of the abdomen showing multiple air-fluid levels.

**Figure 2.**
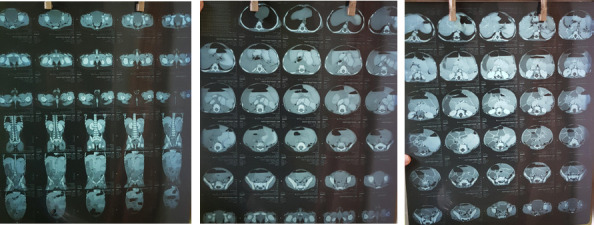
A,B,C. CECT findings were compatible with congenital bands and small bowel strictures.

The child was managed with Ondansetron and fluid replacement which decreased the frequency of vomiting. He was treated as a case of acute viral enteritis. But, considering the severity of the vomiting and the high number of episodes with which he presented, and because of the concern of the parents, a plain abdominal radiograph was sent. The X-Ray findings changed the diagnosis as well as the course of treatment entirely because it showed dilated loops of the small bowel with multiple air-fluid levels (Figure 1). A nasogastric tube was inserted. To find out the cause of obstruction producing such X-Ray findings, a contrast-enhanced computed tomography (CECT) of the abdomen and pelvis was done under intravenous sedation. The CECT showed a distended stomach and dilated duodenum, jejunum and proximal ileum loops (Figure 2 A,B,C). Most of the colon segments were collapsed and a few mildly enlarged mesenteric lymph nodes were seen. It was compatible with congenital bands and small bowel strictures. Following these investigations, the child was kept in non per os and given intravenous hydration.

A surgery referral was made and he underwent surgery. During which, malrotation of the gut was noted. The ileal segment was narrowed. Congenital bands were seen 35cm proximal to the ileocecal junction and were transected. There was no ischemia or peritonitis. The child was put under postoperative observation in the high care unit for 11 days.

During his postoperative period, he gradually recovered and tolerated oral feed after 9 days of stay at the high care unit. He was shifted to the ward for 2 days and discharged with prescription of syrup Flexon 5ml per oral three times a day for three days.

## DISCUSSION

We reported a rare case of intestinal obstruction in a child, which was strongly evoked by history, and radiological findings. Although initially thought to be a viral enteritis, radiologic investigations changed the course of treatment and outcome for the patient. Had a diagnosis not been made on time, or had the patient been discharged after symptomatic relief by antiemetics, possible severe complications of intestinal obstruction could have caused the patient to land up in a worse condition.

In the reported cases, the diagnosis of congenital bands was made intraoperatively.^2,5^ Although we didn't make a diagnosis of congenital bands via CECT, we did suspect it to be the cause. However, a definitive diagnosis was made operatively. Laparoscopy was also rarely used in the reported cases.^5^ But, laparoscopic surgery would have allowed a relatively safer and effective treatment.^4^

We have concluded that mechanical obstruction due to congenital bands must also be considered as a differential when children present with acute onset of excessive vomiting. A plain radiograph of the abdomen must be sent whenever in doubt and CECT must be performed to establish a diagnosis. Laparoscopy would have been a feasible, safe and effective treatment for obstructions caused by congenital bands.^6^

## Consent:

**JNMA Case Report Consent Form** was signed by the patient and the original article is attached with the patient's chart.

## Conflict of Interest

**None.**
